# The role of the fornix in human navigational learning

**DOI:** 10.1016/j.cortex.2019.10.017

**Published:** 2020-03

**Authors:** Carl J. Hodgetts, Martina Stefani, Angharad N. Williams, Branden S. Kolarik, Andrew P. Yonelinas, Arne D. Ekstrom, Andrew D. Lawrence, Jiaxiang Zhang, Kim S. Graham

**Affiliations:** a Department of Psychology, Royal Holloway University of London, Egham, UK; b Cardiff University Brain Research Imaging Centre, School of Psychology, Cardiff University, Cardiff Wales, UK; c Center for the Neurobiology of Learning & Memory, University of California, Irvine, USA; d Department of Psychology, University of California, Davis, CA, USA; e Center for Neuroscience, University of California, Davis, CA, USA; f Department of Psychology, The University of Arizona, AZ USA

**Keywords:** Hippocampus, Spatial navigation, Spatial learning, Episodic memory, Diffusion MRI

## Abstract

Experiments on rodents have demonstrated that transecting the white matter fibre pathway linking the hippocampus with an array of cortical and subcortical structures - the fornix - impairs flexible navigational learning in the Morris Water Maze (MWM), as well as similar spatial learning tasks. While diffusion magnetic resonance imaging (dMRI) studies in humans have linked inter-individual differences in fornix microstructure to episodic memory abilities, its role in human spatial learning is currently unknown. We used high-angular resolution diffusion MRI combined with constrained spherical deconvolution-based tractography, to ask whether inter-individual differences in fornix microstructure in healthy young adults would be associated with spatial learning in a virtual reality navigation task. To efficiently capture individual learning across trials, we adopted a novel curve fitting approach to estimate a single index of learning rate. We found a statistically significant correlation between learning rate and the microstructure (mean diffusivity) of the fornix, but not that of a comparison tract linking occipital and anterior temporal cortices (the inferior longitudinal fasciculus, ILF). Further, this correlation remained significant when controlling for both hippocampal volume and participant gender. These findings extend previous animal studies by demonstrating the functional relevance of the fornix for human spatial learning in a virtual reality environment, and highlight the importance of a distributed neuroanatomical network, underpinned by key white matter pathways, such as the fornix, in complex spatial behaviour.

## 1 Introduction

The ability to navigate, and learn the location of rewards and goals in the environment, is a fundamental and highly adaptive cognitive function across motile species (Ekstrom et al., 2018, Landau and Lakusta, 2009, Murray et al., 2016, Poulter et al., 2018). Lesion studies in animals suggest that this ability depends, in part, on several key brain regions, including the hippocampus (or its non-mammalian homologue), mammillary bodies, and the anterior thalamic nuclei (Murray et al., 2016, Sutherland and Rodriguez, 1989, Warburton and Aggleton, 1998), which in turn connect with a broader network including prefrontal, entorhinal, parahippocampal, retrosplenial, and posterior parietal cortices, all thought to be important for navigation (Ekstrom et al., 2017, Epstein et al., 2017). In particular, these distributed brain structures are connected anatomically by a prominent, arch-shaped white matter pathway called the fornix, which projects from the subiculum and CA1 of the hippocampus toward medial diencephalon, prefrontal cortex and ventral striatum Cenquizca & Swanson, 2007, Saunders and Aggleton, 2007. Given the role of these interconnected structures in spatial learning and navigation (Goodroe et al., 2018, Hunsaker and Kesner, 2018, Ito, 2018, Jankowski et al., 2013), the ability for these distributed regions to communicate via the fornix may also be critical for successful spatial learning and navigation, as predicted by network-level accounts of the neural substrates of human spatial abilities (Ekstrom et al., 2017, Hinman et al., 2018).

The Morris Water Maze (MWM) is one of the most widely used laboratory tasks in studies of navigational behaviour across non-human species and has been recognised as an excellent candidate for a universal test of spatial navigation ability Morris, 2015, Morris et al., 1982, Possin et al., 2016. In this task, rodents are placed in a circular pool and required to swim to a hidden platform beneath the surface using cues outside the pool. Several studies have shown that fornix-transected rodents are impaired in learning this task, particularly when required to navigate flexibly from multiple positions within the maze (Cain et al., 2006, De Bruin et al., 2001, Eichenbaum et al., 1990, Packard and McGaugh, 1992, Warburton and Aggleton, 1998, Warburton et al., 1998). Fornix transection also impairs place learning in other maze-based tasks (Dumont et al., 2015, Hudon et al., 2003, Olton et al., 1978, O'Keefe et al., 1975, Packard et al., 1989).

Critically, while these rodent studies highlight a key role for the fornix in spatial learning across a range of visuospatial and navigation tasks, the role of this white matter pathway in human wayfinding is currently unknown. Studies using diffusion magnetic resonance imaging (dMRI), which allows the *in vivo* reconstruction of white matter fibre pathways and insights into their microstructural properties Assaf, Johansen-Berg, & Thiebaut de Schotten, 2019, Wandell, 2016 have reported associations in healthy human participants between fornix microstructure and inter-individual differences in episodic memory (Bennett et al., 2015, Hodgetts et al., 2017, Rudebeck et al., 2009). As is the case for episodic memory (Palombo, Sheldon, & Levine, 2018), there are marked inter-individual differences in navigational abilities and spatial functioning (Weisberg and Newcombe, 2018, Wolbers and Hegarty, 2010). A key question is whether inter-individual differences in human navigation ability are related to inter-individual differences in fornix microstructure.

**Fig. 1:**
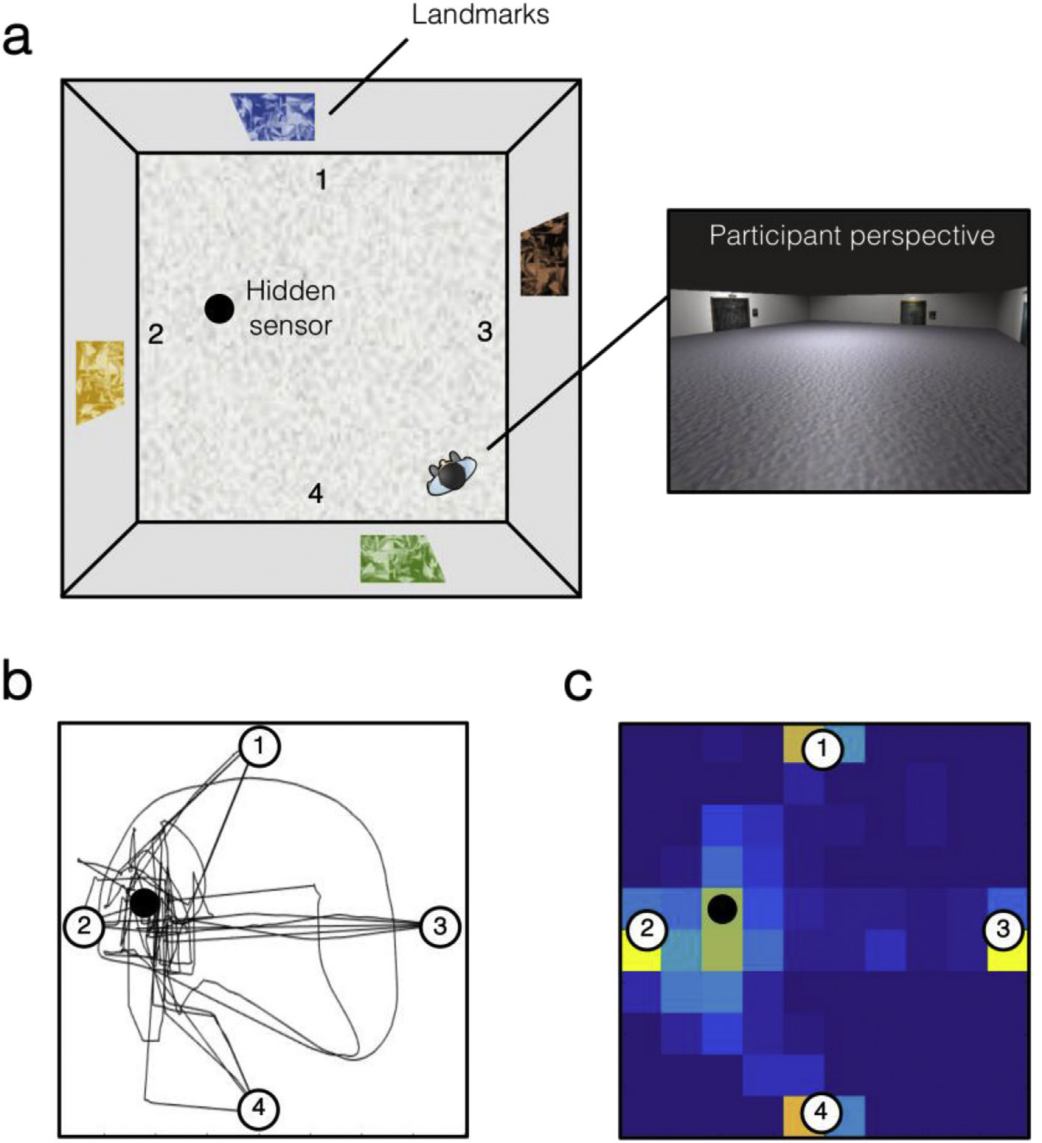
The virtual reality navigational learning task based on the Morris Water Maze. (A) Birds-eye schematic of the virtual art gallery that the participants explore during the task. The artworks on the outer walls of the gallery are the “landmarks” in the virtual arena. An example first person perspective from within the maze is shown. (B) Movement trajectories and (C) Location heatmap across all 20 trials for an example participant.

To examine this question, we acquired dMRI data in healthy human participants who performed a virtual-reality navigational learning task based on the MWM, wherein individuals were required to learn, over trials, the location of a hidden sensor within a virtual arena. Similar to classic rodent paradigms, such as the MWM, participants were required to navigate from multiple starting positions across trials, thus placing greater demand on flexible allocentric (‘map-like’) or relational processing (Fig. 1) (Eichenbaum et al., 1990, Morris et al., 1982). To create a single index of navigational learning rate, we used a curve fitting approach to model the time taken to reach the sensor across trials (for similar approaches, (see Kahn et al., 2017, Pereira and Burwell, 2015, Stepanov & Abramson, 2008]. We predicted that fornix microstructure would be significantly related to spatial learning rate in our navigational learning task. As a comparison tract, we selected the inferior longitudinal fasciculus (ILF) - a major white matter bundle connecting occipital with anterior temporal regions (Catani et al., 2003, Latini, 2015). Previous dMRI studies have shown that this tract is less associated with performance on episodic memory tasks, and may be more strongly linked to visual object and semantic processing (Herbet et al., 2018, Hodgetts et al., 2017, Hodgetts et al., 2015), including semantic learning (Ripollés et al., 2017). In addition, studies in rodents suggest that lesions to putatively homologous object processing pathways do not impair spatial learning in the MWM (Burwell et al., 2004, Bussey et al., 1999). We therefore predicted that ILF microstructure would be unrelated to spatial learning rate.

## 2 Methods

### 2.1 Participants

Thirty-three healthy volunteers (18 females; 15 males; mean age = 24 years; SD = 3.5 years; range = 19–33) were scanned at the Cardiff University Brain Research Imaging Centre (CUBRIC) using diffusion-weighted MRI. These same participants completed a virtual navigation task in a separate behavioural session conducted at a later date (average time delay between sessions: ~6 months; range = 28–339 days). All participants were fluent English speakers with normal or corrected-to-normal vision. Participation in both sessions was undertaken with the understanding and written consent of each participant. The research was completed in accordance with, and approved by, the Cardiff University School of Psychology Research Ethics Committee.

### 2.2 Virtual Morris Water Maze task

We used the virtual MWM task developed by Kolarik et al. (2016). This task was created using Unity 3D (Unity Technologies, San Francisco) and required participants to explore, from a first-person perspective, a virtual art gallery using the arrow keys on the computer keyboard (Fig. 1A). The room was 8 × 8 virtual m^2^ in size, and contained four distinct paintings, one on each wall of the environment. On a given trial, the participants' task was to locate a hidden sensor on the floor as quickly as possible. This sensor occupied .25% of the total floor space (i.e., an .8 × .8 m^2^ square). When the participant walked over the hidden platform it became visible and the caption ‘You found the hidden sensor’ was displayed in the centre of the screen. At this point, the exploration time was recorded automatically and a 10 sec countdown appeared in the centre of the display during which the participants could freely navigate the room. After this countdown, an inter-trial window appeared and the participants could click on a button to start the next learning trial. The maximum duration of each learning trial was 60 sec. If the participant did not find the target location within this period, the sensor became visible. The task involved 20 learning trials, which comprised five blocks of four trials. The blocked structure was not made explicit to the participant (i.e., there was no break every four trials). Each trial within a block began at a different, randomly-selected starting position within the environment (arbitrary ‘North’, ‘South’, ‘East’, or ‘West’). The same random trial order was used across all participants. The movement trajectories and location heatmap for an example participant are shown in Fig. 1B, C.

### 2.3 MRI acquisition

Whole brain dMRI data were acquired at the Cardiff University Brain Research Imaging Centre (CUBRIC) using a 3T GE HDx Signa scanner with an eight-channel head coil. Single-shell high-angular resolution dMRI (HARDI) (Tuch et al., 2002) data were collected with a single-shot spin-echo echo-planar imaging pulse sequence with the following parameters: TE = 87 msec; voxel dimensions = 2.4 × 2.4 × 2.4 mm^3^; field of view = 23 × 23 cm^2^; 96 × 96 acquisition matrix; 60 contiguous slices acquired along an oblique–axial plane with 2.4 mm thickness (no gap). Gradients were applied along 30 isotropic directions with b = 1200 sec/mm^2^. Three non-diffusion weighted images were acquired with b = 0 sec/mm^2^. The scans were cardiac-gated using a peripheral pulse oximeter placed on the participants' fingertips. A T1-weighted 3D FSPGR sequence was also acquired with the following parameters: TR = 7.8 msec; TE = 3 msec, TI = 450 msec, flip angle = 20°; FOV = 256 mm*192 mm*172 mm; 1 mm isotropic resolution.

### 2.4 Diffusion MRI preprocessing

Diffusion MRI data were corrected for participant head motion and eddy currents using ExploreDTI (Version 4.8.3; Leemans & Jones, 2009). The bi-tensor ‘Free Water Elimination’ (FWE) procedure was applied *post hoc* to correct for voxel-wise partial volume artifacts arising from free water contamination (Pasternak, Sochen, Gur, Intrator, & Assaf, 2009). Free water contamination (from cerebrospinal fluid) is a particular issue for white matter pathways located near the ventricles (such as the fornix), and has been shown to significantly affect tract delineation (Concha, Gross, & Beaulieu, 2005). Following FWE, corrected diffusion-tensor indices FA and MD were computed. FA reflects the extent to which diffusion within biological tissue is anisotropic, or constrained along a single axis, and can range from 0 (fully isotropic) to 1 (fully anisotropic). MD (10^−3^mm^2^s^−1^) reflects a combined average of axial diffusion (diffusion along the principal axis) and radial diffusion (diffusion along the orthogonal direction). The resulting corrected FA and MD maps were used as inputs for tractography analysis.

### 2.5 Tractography

Deterministic whole brain white matter tractography (Wandell, 2016) was performed using the ExploreDTI graphical toolbox. Tractography was based on constrained spherical deconvolution (CSD) (Dell’Acqua & Tournier, 2019), which can extract multiple peaks in the fibre orientation density function (fODF) at each voxel. This approach permits the representation of bending/crossing/kissing fibres in individual voxels. Each streamline was reconstructed using an fODF amplitude threshold of .1 and a step size of 1 mm, and followed the peak in the fODF that subtended the smallest step-wise change in orientation. An angle threshold of 30° was used and any streamlines exceeding this threshold were terminated.

Three-dimensional reconstructions of each tract were obtained from individual participants by using a waypoint region of interest (ROI) approach, based on an anatomical prescription. Here, “AND” and “NOT” gates were applied, and combined, to extract tracts from each participant's whole brain tractography data. These ROIs were drawn manually on the direction-encoded FA maps in native space by one experimenter (MS) and quality assessed by two other authors (CJH, ANW). After tract reconstructions for each participant, mean FA/MD values were calculated by averaging the values at each 1 mm step along each tract.

#### 2.5.1 Fornix

**Fig. 2:**
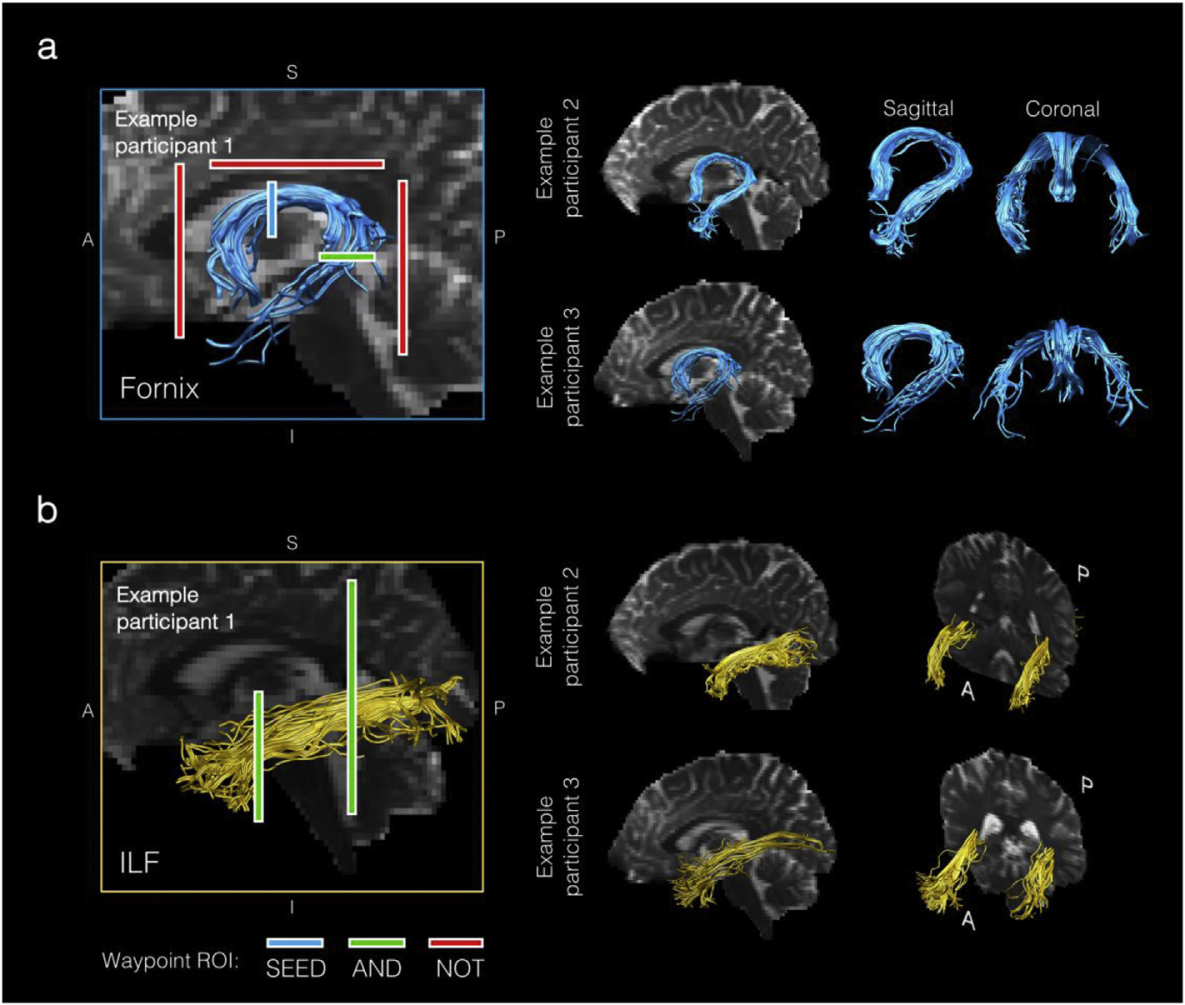
The deterministic tractography protocol for the fornix and ILF. (A) Example reconstructions of the fornix in three participants. The left image shows the placement of waypoint ROIs on a midline non-diffusion-weighted image. The reconstructed fornices from two other participants are shown on the right from sagittal and coronal orientations. (B) Reconstructions of the inferior longitudinal fasciculus (ILF) in the same exemplar participants. As for the fornix, the left image shows the placement of waypoint ROIs on a midline non-diffusion-weighted image. The reconstructed *bilateral* fasciculi from two other participants are shown on the right. The protocol for ROI placement can be found in the main text (Section 2.5).

A multiple region-of-interest (ROI) approach was adopted to reconstruct the whole fornix (Metzler-Baddeley, Jones, Belaroussi, Aggleton, & O’Sullivan, 2011). This approach involved placing a seed point ROI on the coronal plane at the point where the anterior pillars enter the fornix body (Fig. 2). Using a mid–sagittal plane as a guide, a single AND ROI was positioned on the axial plane, encompassing both crus fornici at the lower part of the splenium of the corpus callosum. Three NOT ROIs were then placed: (1) anterior to the fornix pillars; (2) posterior to the crus fornici; and (3) on the axial plane, intersecting the corpus callosum. Once these ROIs were placed, and the tracts reconstructed, anatomically implausible fibers were removed using additional NOT ROIs (see Hodgetts et al., 2017).

#### 2.5.2 Inferior longitudinal fasciculus (ILF)

Fibre-tracking of the ILF (comparison tract) was performed using a two-ROI approach in each hemisphere (Wakana et al., 2007). First, the posterior edge of the cingulum bundle was identified on the sagittal plane. Reverting to a coronal plane at this position, a SEED ROI was placed that encompassed the whole hemisphere. To isolate streamlines extending towards the anterior temporal lobe (ATL), a second ROI was drawn at the most posterior coronal slice in which the temporal lobe was not connected to the frontal lobe. Here, an additional AND ROI was drawn around the entire temporal lobe (Fig. 2). Similar to the fornix protocol above, any anatomically implausible streamlines were removed using additional NOT ROIs. This approach was carried out in each hemisphere (Fig. 2); tract-averaged diffusion metrics for the left and right ILF were averaged to create a bilateral measure of ILF FA and MD in each participant.

### 2.6 Grey matter volumetry

Bilateral hippocampal volume was derived using FMRIB's Integrated Registration & Segmentation Tool (FIRST; Patenaude, Smith, Kennedy, & Jenkinson, 2011). As the volumes of temporal lobe substructures have been shown to correlate with intracranial volume (Moran, Lemieux, Kitchen, Fish, & Shorvon, 2001), individual-level hippocampal volumes were divided by total intracranial volume (eTIV) to create proportional scores (Westman, Aguilar, Muehlboeck, & Simmons, 2013).

### 2.7 Statistical analysis of maze learning

**Fig. 3:**
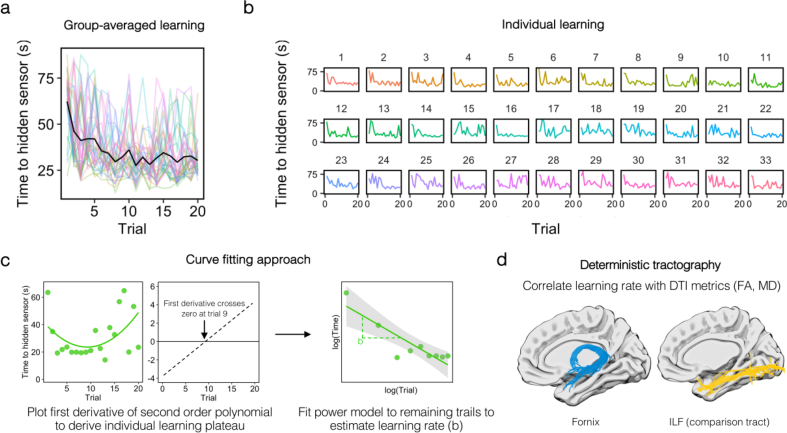
Modelling navigational learning in individual participants. Task learning at the (A) group-level and (B) individual-level. Y-axes represent the time to reach the hidden sensor in seconds. The number of trials (total = 20) is shown on the x-axis. (C) Method for determining the number of learning trials to-be-modelled. Several participants appeared to learn rapidly and plateau before displaying variable performance in later trials. For instance, a power model fits the example participant's latency data poorly when all trials are considered. In order to capture initial learning, therefore, we fitted the latency data (across all trials) with a second-order polynomial in each participant. The point at which the first derivative of this polynomial crossed zero was used to define the number of trials to-be-modelled. The trials up to this point were then fit with a power function and the *b* parameter derived to index learning rate. Power fits are shown by linearly fitting the log-transformed data. (D) Learning rate measures were correlated with diffusion tensor metrics (FA, MD) from the fornix (blue) and the ILF (yellow). Tract reconstructions are shown against an inflated brain for visualisation purposes.

To increase sensitivity to individual-level performance across learning trials, and to derive a single index of learning rate, we analysed the relationship between spatial learning and fornix tissue microstructure using a curve fitting approach (see e.g., Kahn et al., 2017, Pereira and Burwell, 2015). Performance on each learning trial was defined by the time (in seconds) to reach the hidden sensor. As can be seen in Fig. 3A, there was high inter-individual variability in spatial learning, with participants varying in both learning speed and the shape of their learning pattern. Here, individual learning data were fit using a power function: Time to sensor = a * x^b^, where *b* specifies the slope of the fitted power model.

One aspect of this data is that several participants learned quickly (and plateaued) before displaying highly variable, or slow, performance in the later trials (e.g., participants 6, 9, 13, 20 and 27; Fig. 3B). This presents a challenge for a curve fitting approach across all trials (and potentially produces counterintuitive results), as some of the fastest learners will show the poorest model fits. For instance, both participants 9 and 16 display an initial steep learning curve and an early plateau (Fig. 3B), but a power model fit to *all trials* provides a poor fit of the participant who does not sustain performance until the end of the task. In order to dissociate learning from potential task motivational factors, we adopted a data-driven approach to determine a cut-off in individual participants prior to our main analysis. Specifically, a second-order polynomial model was fit to all trials in each participant using the curve fitting toolbox in Matlab (Mathworks, Inc.). The cut-off was defined as the trough of this curve, which is where the first derivative of the second-order polynomial crosses zero (Fig. 3C). Trials up to and including this cut-off were then modelled using a power function (mean trials included = 14.3; range = 7–20).

Using this approach, we derived a single index of learning rate, denoted by the *b* parameter (or slope) of the fitted power model (*b*; mean = −.32, SD = .08, range = −.49 to −.19). The *b* parameter reflects slope curvilinearity in each participant, where lower, negative values reflect more convex downward curves and thus faster learning rates. Since higher FA/lower MD is typically associated with microstructural properties that support the efficient transfer of information along white matter tracts (Beaulieu, 2002), at least in adults, we predicted a positive association between fornix MD and learning rate, and negative associations between fornix FA and learning rate.

Directional Pearson correlations (Lakens, 2016) were conducted between the learning rate (*b*) and free water corrected MD and FA values for the fornix and ILF (Fig. 2, Fig. 3). The resulting coefficients were compared statistically using directional Steiger Z-tests (Steiger, 1980) within the ‘cocor’ package in R (Diedenhofen & Musch, 2015). Pearson correlations were Bonferroni-corrected by dividing α = .05 by the number of statistical comparisons for each DTI metric (i.e., .05/2 = .025) (Lakens, 2016). Rather than use an arbitrary cut-off to exclude poor performers on the task, we instead used a data-driven resampling approach where each individual's trial-wise latencies were shuffled over 500 permutations. For each random permutation, we fitted a power function to the data and derived an R^2^ to evaluate model fit. Participants with a true R^2^ (i.e., based on their actual performance) that fell below the 68% CI of their individually-defined random distribution (i.e., 1 SD) were excluded (participants 10, 15, 17, 18 and 21; Analysed: N = 28 [16 females; 12 males]). Prior to correlational analyses, outliers for each tract metric were identified and removed using 2.5 median absolute deviations (MAD) (see Leys et al., 2013, Leys et al., 2019 for a discussion of this approach). This excluded an outlier value for fornix MD (Analysed: N = 27 [16 females; 11 males]) and 2 for fornix FA (Analysed: N = 26 [15 females; 11 males]).

Additional Bayesian correlation analyses were conducted using JASP (https://jasp-stats.org). From these, we report default Bayes factors and 95% Bayesian credibility intervals (BCI). The Bayes factor, expressed here as either BF_+0_ or BF_-0_ grades the intensity of the evidence that the data provide for the alternative hypothesis (H1) versus the null (H0) on a continuous scale. BF_+0_ refers to the predicted positive association between our behavioural measures and mean diffusivity, and BF_-0_ denotes the predicted negative association with FA (see above). BF of 1 indicates that the observed finding is equally likely under the null and the alternative hypothesis. A BF_+0/-0_ much greater than 1 allows us to conclude that there is substantial evidence for the alternative over the null. Conversely BF_+0/-0_ values substantially less than 1 provide strong evidence in favour of the null over the alternative hypothesis (Wetzels & Wagenmakers, 2012).

Frequentist and Bayesian partial correlations were carried out using ‘ppcor’ (Seongho, 2015) and ‘BayesMed’ (Wetzels & Wagenmakers, 2012) packages in R, respectively. Complementary Spearman's rho tests were also conducted for our key correlations. The magnitudes of Spearman's correlations were compared directly using a robust bootstrapping approach (Wilcox, 2016). This was performed using the Robust Correlation Toolbox (Pernet, Wilcox, & Rousselet, 2013) and ‘comp2dcorr’ in Matlab (https://uk.mathworks.com/) (https://github.com/GRousselet/blog/tree/master/comp2dcorr).

## 3 Results

### 3.1 Correlating navigational learning with tract microstructure

**Fig. 4:**
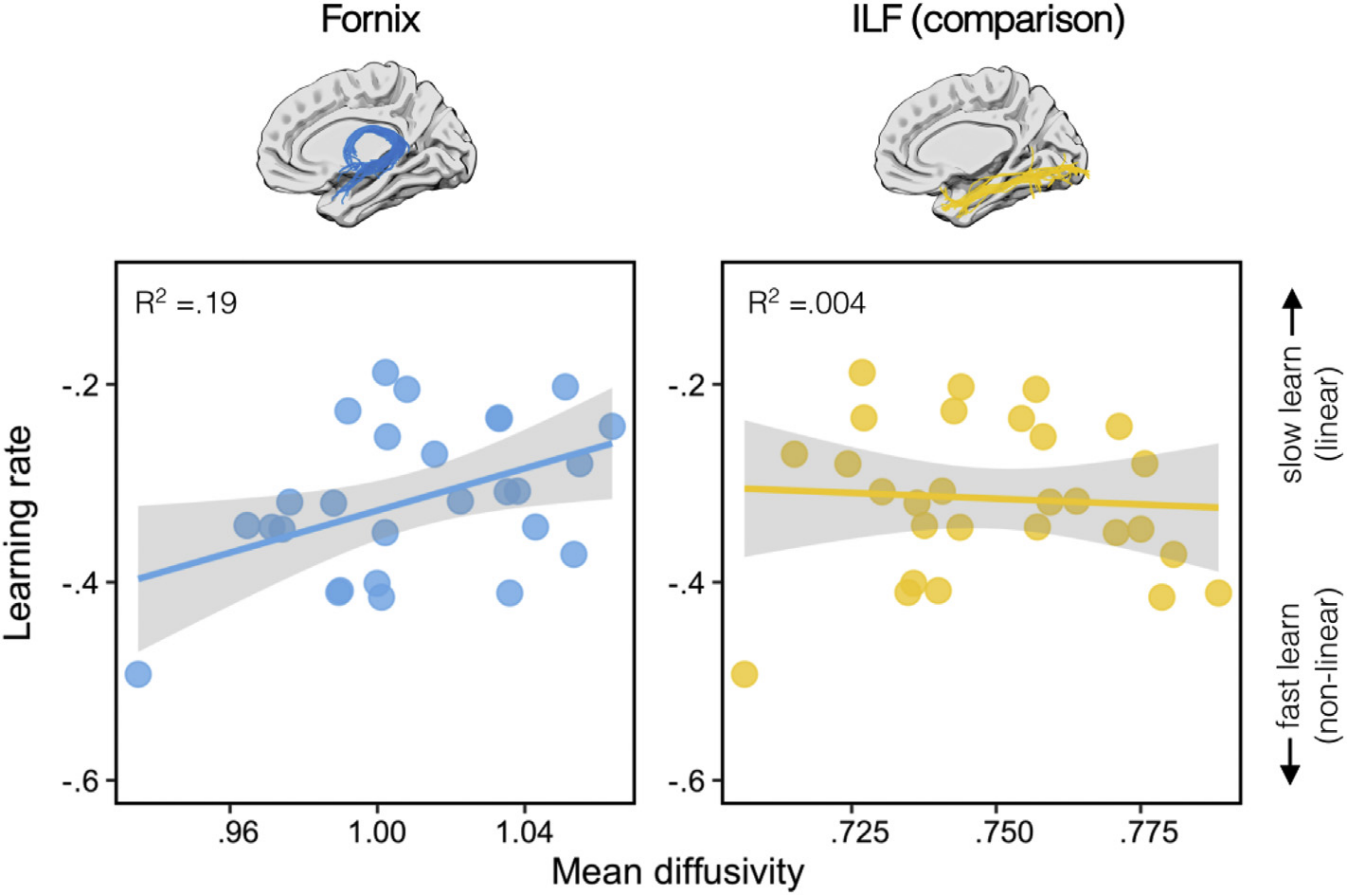
The correlation between mean diffusivity (MD) and learning rate (*b* parameter) for the fornix (left) and the inferior longitudinal fasciculus (right).

There was a significant positive correlation between the derived learning rate (*b*) and fornix MD, as shown in Fig. 4. This suggests that those participants with lower fornix MD had faster learning rates (r = .44, *p* = .01, 95% BCI [.09, .68], B_+0_ = 5.46; Fig. 4). There was no significant association between individual learning rate and MD in a comparison tract - the inferior longitudinal fasciculus (ILF; r = −.07; *p* = .63, 95% BCI [.37, .01], B_+0_ = .19). A directional Steiger Z-test revealed that the correlation between derived learning rate and fornix MD was significantly greater than with ILF MD (z = 2.26, *p* = .01).

A medium–size correlation was observed between fornix FA and learning rate but this did not reach significance (r = −.24, *p* = .09, 95% BCI [-.56, −.02], B_-0_ = .86). There was no significant correlation between ILF FA and learning rate (r = −.15; *p* = .22, 95% BCI [-.49, −.01], B_-0_ = .48). These two correlations did not differ significantly (z = .13, *p* = .45).

### 3.2 Controlling for hippocampal volume

To examine whether hippocampal volume contributes to the microstructural–behavioural correlations reported above, partial correlations (both frequentist and Bayesian) were conducted. The significant positive correlation between the learning rate parameter and fornix MD remained when controlling for bilateral hippocampal volume (r = .41, *p* = .02, BF_+0_ = 3.88) (see also Hodgetts et al., 2017). Partialing out hippocampal volume did not strongly influence the moderate association between fornix FA and learning rate (r = −.24, *p* = .13, BF_-0_ = .79). When examining whether hippocampal volume was negatively associated with *b*, independent of fornix microstructural measures, there was no significant association between hippocampal volume and learning rate (*b*) (r = .3, *p* = .94, 95% BCI [-.25, −.002], B_-0_ = .1).

### 3.3 Non-parametric correlations between tract microstructure and learning

Finally, we also conducted complementary directional Spearman's rho tests for our key correlations, with such tests robust to univariate outliers (Croux and Dehon, 2010, Winter et al., 2016). As above, Spearman's correlations were Bonferroni-corrected by dividing α = .05 by the number of statistical comparisons for each DTI metric (i.e., .05/2 = .025). A significant positive association was observed between learning rate and fornix MD (ρ = .4, *p* = .02). No significant association was found with ILF MD (ρ = −.18, *p* = .82). A moderate correlation was observed between the *b* parameter and fornix FA (ρ = −.26, *p* = .1), which was lower for ILF FA (ρ = −.1, *p* = .29).

A direct comparison between these correlations revealed a significant difference between fornix MD and ILF MD and their association with navigation learning rate, as indicated by the bootstrap distribution not overlapping with zero (95% CI = [.22, .91]). There was no significant difference between the FA correlations (95% CI = -[.63, .34]).

### 3.4 Supplementary post-hoc analyses

#### 3.4.1 The influence of gender on brain-behaviour correlations

Based on prior work showing spatial navigation differences between males and females Coutrot et al., 2018, Wolbers and Hegarty, 2010, we conducted an additional *post-hoc* analysis to examine whether our main result remains when controlling for gender. Using a partial correlation approach, as above, we found that the significant relationship between fornix MD and *b* was maintained when controlling for participant gender (r = .38, *p* = .03, BF_+0_ = 2.64).

#### 3.4.2 Correlating mean latency with tract microstructure

**Fig. 5:**
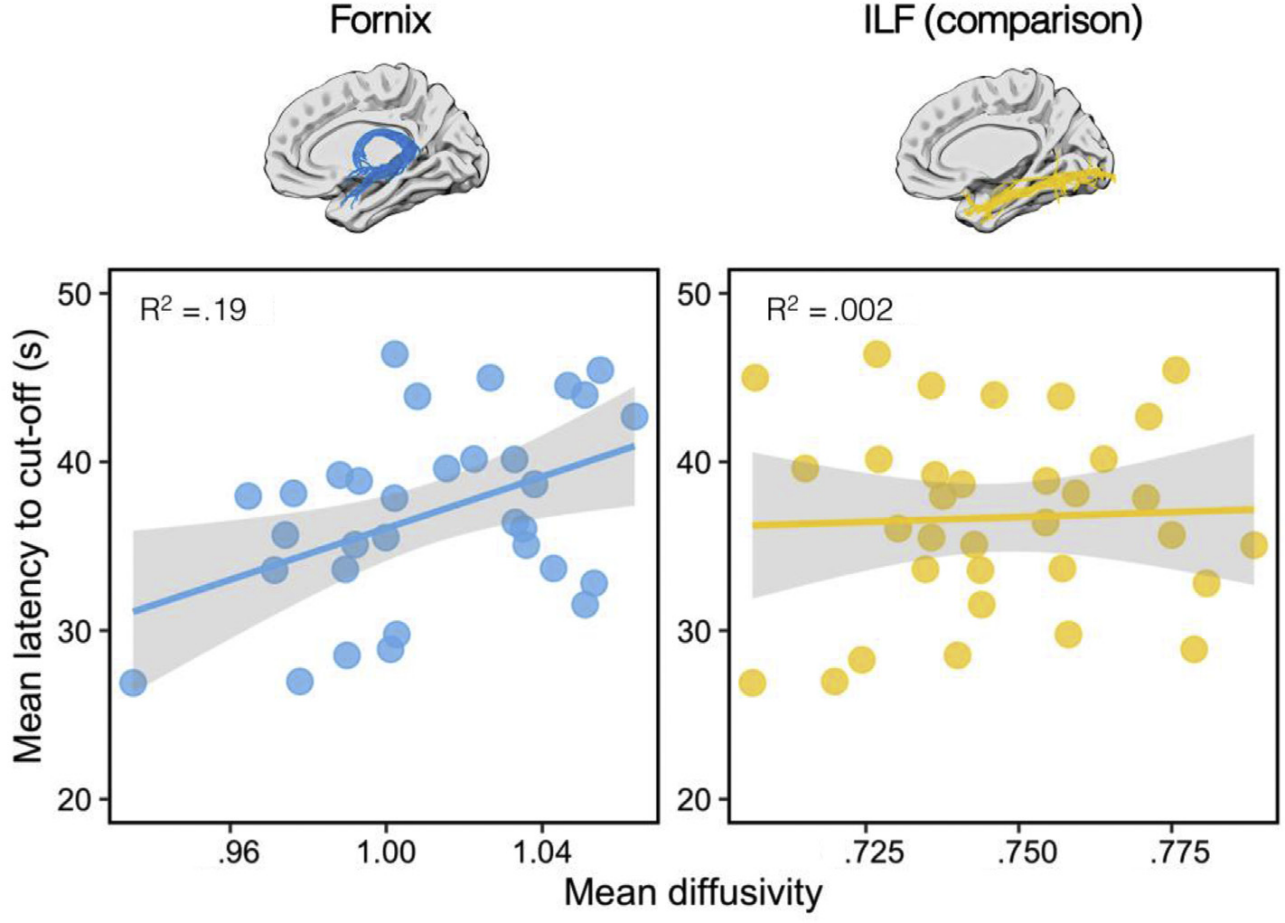
The correlation between mean diffusivity (MD) and mean latency to the hidden sensor (averaged from Trial 1 to the cut-off) for the fornix (left) and the inferior longitudinal fasciculus (right).

As described in the Methods Section 2.7, a subset of participants was excluded from our main analysis as they did not show robust behavioural evidence of learning in our task. To conduct an analysis that incorporates these participants, we derived an alternative non-slope-based measure of performance: *mean latency to the cut-off*. While this may be less sensitive to information inherent in the learning curve, this method should still discriminate between participants who differ in overall levels of performance (i.e., participants who are consistently fast vs. slow). As can be seen in Fig. 5, we find a strong positive association between fornix MD and mean latency (r = .44, *p* = .006, 95% BCI [.11, .67], B_+0_ = 9). There was no significant association between ILF MD and mean latency (r = .04, *p* = .4, 95% BCI [.01, .4], B_+0_ = .27). There was a difference between these correlations at the *p* < .1 level (Z = 1.29, *p* = .09).

As with our learning rate measure, above, there were no significant associations between mean latency and FA for either white matter tract (fornix: r = −.02, *p* = .46, 95% BCI [-.4, −.01], B_-0_ = .24; ILF: r = .04, *p* = .4, 95% BCI [-.43, −.01], B_-0_ = .33).

## 4 General discussion

Using a virtual reality (VR) paradigm modelled on the Morris Water Maze, we examined whether inter-individual differences in the microstructure of the human fornix, a white matter pathway linking hippocampus with an array of cortical and subcortical structures, are related to inter-individual differences in flexible navigational learning. To increase sensitivity to individual learning across trials we adopted a curve fitting approach (Kahn et al., 2017), which generated a single index of learning rate for each individual. We found that fornix microstructure (particularly mean diffusivity, MD) was significantly associated with navigational learning rate, as defined by the slope of the fitted power model (*b*), such that those participants with lower fornix MD had faster learning rates, and this association remained significant when controlling for bilateral hippocampal volume. Furthermore, this correlation was significantly stronger than that seen for the ILF, a comparison tract linking occipital and anterior temporal cortices, which has previously been implicated in complex object processing and semantic learning Herbet et al., 2018, Hodgetts et al., 2015, Postans et al., 2014, Ripollés et al., 2017.

These results build upon, and extend, previous animal studies that highlight a potential key role for the fornix (but not visual object processing pathways) in mediating flexible place learning and navigational behaviour. Critically, we provide novel evidence, using a virtual reality MWM task similar to that used in animals (Kolarik et al., 2016, Possin et al., 2016), that the fornix supports navigational learning in humans. In rodents, fornix transection has been shown to impair MWM learning, as characterised by more gradual learning slopes and slower latencies in finding the hidden platform (Cain et al., 2006, Eichenbaum et al., 1990, Packard and McGaugh, 1992, Warburton and Aggleton, 1998). Indeed, in one study fornix transection was shown to impair learning while probe trial performance was unaffected (Warburton & Aggleton, 1998). By applying a curve fitting approach, we were able to characterise the steepness of learning slopes at the individual participant level, and relate this directly with fornix microstructure. Strikingly consistent with the animal studies described above, reduced microstructural integrity in the fornix (indexed by higher MD) was associated with more gradual spatial learning rates. Further, by identifying individual learning plateaus in a data-driven way, our approach also accounts for potential fatigue, mind-wandering or other factors that may affect performance later in the learning session.

Similar to the effects of lesioning the hippocampus (Morris et al., 1982) and anterior thalamic nuclei (Warburton & Aggleton, 1998), learning deficits following fornix transection in rodents are most pronounced when the animal is required to navigate from multiple start positions (Eichenbaum et al., 1990), or when extra-maze landmarks are rotated on each trial (Hudon et al., 2003). Such findings suggest, therefore, that this broader, extended hippocampal system supports the acquisition of flexible spatial representations based on the relationship between the goal and environmental landmarks (Eichenbaum et al., 1990). This is in contrast to response or route-based learning from a particular start or vantage point, which appears recruit regions outside the extended hippocampal system, such as the caudate nucleus (Chersi and Burgess, 2015, Devan et al., 1996, Hinman et al., 2018, Packard and McGaugh, 1992). Consistent with this, we observed an association between navigational learning (learning rate and mean latency) and fornix microstructure in a task that required participants to navigate to the goal from multiple starting positions (and presumably required the ability to use distal landmarks to navigate).

The similarity between our findings and those in rodents is particularly striking given that desktop virtual reality navigation (i.e., from a stationary sitting position) does not provide idiothetic, self-motion cues (Starrett & Ekstrom, 2018), which are important inputs to hippocampal place fields in rodents (Sharp, Blair, Etkin, & Tzanetos, 1995; but see Chen, Lu, King, Cacucci, & Burgess, 2019, on VR navigation in rodents). Humans and other primates rely much more than rodents on detailed vision for spatial navigation (Ekstrom, 2015). The primate hippocampus contains view-coding cells (Rolls & Wirth, 2018), which might be particularly relevant for VR-based navigation (Ekstrom, 2015). Nevertheless, our findings suggest that the mechanisms underpinning virtual reality navigation and real world navigation share a great deal in common.

Overall, this study provides support for the idea that an individual's spatial navigation ability (Weisberg and Newcombe, 2018, Wolbers and Hegarty, 2010) is underpinned, at least in part, by the integrated functioning of a distributed neuroanatomical network, comprising not only individual regions (such as the hippocampus and anterior thalamic nuclei), but also the white matter connections linking these brain areas (i.e. the fornix, together with non-fornical connections) (Jankowski et al., 2013, Murray et al., 2016). This view does not necessitate that the role of the fornix in network communication is identical to that of any of the individual regions it connects (Wandell, 2016). For instance, while fornix transection impairs, or at least slows, navigational learning in the MWM (Warburton & Aggleton, 1998), as discussed above, these impairments are not as severe as those seen following lesions to the anterior thalamic nuclei or the hippocampus proper (Cain et al., 2006, Eichenbaum et al., 1990, Ikonen et al., 2002, Warburton and Aggleton, 1998) - despite fornix transection having widespread impact on a network of structures normally activated by spatial memory processes (Vann, Brown, Erichsen, & Aggleton, 2000). This is not to suggest that fornix connectivity is not important for place representations Miller and Best, 1980, Shapiro et al., 1989, but rather that the fornix may support processes which help build, support and flexibly deploy detailed cognitive maps in conjunction with other brain areas involved in a broader distributed navigation network (Ekstrom et al., 2017, Hinman et al., 2018). For instance, microstructural properties of the fornix may support synchronised functional coupling between distal brain regions by regulating conduction velocities Bechler et al., 2018, Bells et al., 2017.

As mentioned in the introduction, previous dMRI studies in humans have reported associations between fornix microstructure and episodic memory (Bennett et al., 2015, Rudebeck et al., 2009), notably the ability to retrieve spatiotemporal detail in real-world memories (Hodgetts et al., 2017). A number of authors have suggested that the extended-hippocampal network's navigational functions, such as the ability to form cognitive maps, supports a derived role in scaffolding episodic memory (Burgess et al., 2002, Lisman et al., 2017, O'Keefe and Nadel, 1976). Relational Memory Theory, by contrast, posits that while the extended hippocampal system is essential to spatial navigation via a cognitive map, its role derives from the relational organisation and flexibility of cognitive maps and not from a foundational role in the spatial domain (Eichenbaum, 2017; see also; Ekstrom & Ranganath, 2017). While our findings do not adjudicate between these accounts, they provide novel evidence of links between the extended hippocampal system and both cognitive mapping and episodic memory in humans.

Note, it is possible that some inter-individual differences in navigational performance may actually reflect differences in types of spatial strategies employed (Weisberg & Newcombe, 2018). For instance, while some individuals may use a strategy akin to cognitive mapping, i.e., based on allocentric vectors from the “landmarks” to the hidden sensor, some individuals may use a strategy based on matching and integrating disparate viewpoints from the sensor location; a strategy more akin to building a model of the broader scene and layout (Wolbers & Wiener, 2014). While participants were not asked about their use of spatial strategies in the current study, this would be an interesting avenue for future large-scale studies to explore, either via subjective ratings or through the application of unbiased machine-learning algorithm to classify distinct spatial strategies (e.g., Illouz et al., 2016). In this context, it would also be interesting to apply the curve-fitting approach outlined here to other measures of navigational behaviour. While search latency (i.e., the time taken to find the goal location) is the most commonly used metric in both human and animal studies of maze learning, there are other possible metrics that may provide additional information about how individuals navigate the maze. For instance, prior work suggests that hippocampal damage may impair the ‘precision' of search trajectories, such that patients search the correct quadrant of the arena but spend less time in the immediate area of the hidden goal (Kolarik et al., 2016, Kolarik et al., 2018). Measures that take into account distance-to-the-goal along search trajectories may be more sensitive to precise spatial behaviour relative to search latencies alone (Gallagher, Burwell, & Burchinal, 1993).

While our findings support the notion that an extended hippocampal-based system, inter-connected by the fornix, may be important for navigational learning in humans, it was notable that the association between fornix microstructure and learning was present when controlling for HC volume. Further, there was strong evidence *against* an association between place learning and HC volume in this task, with the BF strongly favouring the null hypothesis. This aligns with our previous finding that fornix microstructure (but not hippocampal volume) predicts individual differences in remembering spatiotemporal aspects of autobiographical memories (Hodgetts et al., 2017). Though some studies have found associations between hippocampal grey matter volume and navigational ability in healthy adults Bohbot et al., 2007, Chrastil et al., 2017, Hao et al., 2016, Hartley and Harlow, 2012, Schinazi et al., 2013, Sherrill et al., 2018, Woollett and Maguire, 2011, recent studies utilising larger samples have failed to do so (Weisberg, Newcombe, & Chatterjee, 2019). In addition, studies of individuals with profound orientation deficits (termed development topographical disorientation, or DTD) similarly show altered hippocampal connectivity (in this case, between hippocampus and medial prefrontal cortex). Interestingly, like in our study, hippocampal grey matter does not appear to explain these differences (Iaria and Barton, 2010, Iaria et al., 2009). This highlights that variation in broader neuroanatomical systems, rather than regional volumetric variation, may be particularly sensitive to inter-individual differences in navigational learning.

Our study has some limitations that will need to be addressed in future work. While the sample size used in the present study is typical, and in fact larger, than many similar investigations of individual differences in navigational behaviour, it will be important to conduct larger-scale confirmatory investigations in the future that will allow more detailed analysis of search strategies and other individual difference factors that may contribute to performance in this task (e.g., gender, age, navigation expertise, etc.) Coutrot et al., 2018, Weisberg, Newcombe, & Chatterjee, 2019. Note, this issue is partly mitigated by a clear hypothesis-driven tract of interest approach (Button et al., 2013) and Bayesian analyses showing that our findings have substantial evidential value (Dienes, 2014).

Similar to our previous work on scene discrimination and episodic memory, we observed stronger effects for fornix MD versus FA (Hodgetts et al., 2015, Postans et al., 2014). The biological interpretation of this difference is not straightforward, as variation in either measure could arise from multiple aspect(s) of the underlying white matter, including axon density, axon diameter, myelination, and the manner in which fibres are arranged in a voxel (Beaulieu, 2002, Wandell, 2016). This is also consistent with reports that FA shows greater intra-tract variability than MD, that is, tracts do not have a signature FA value that is consistent along the tract length (Yeatman, Dougherty, Myall, Wandell, & Feldman, 2012). It is possible, therefore, that MD may be a more ‘tract representative’ measure, and thus better suited to tractography approaches that involves averaging along white matter pathways. A recent study reported strong correspondence between DTI microstructural indices and underlying tissue microstructure, where high FA was linked to high myelin density and a sharply tuned histological orientation profile, whereas high MD was related to diffuse histological orientation and low myelin density (Seehaus et al., 2015). Diffusion MRI studies applying more advanced biophysical models of white matter microstructure may be able to provide additional insight into the specific biological attributes underlying these brain-behaviour associations Assaf, Johansen-Berg, & Thiebaut de Schotten, 2019, Karahan, Costigan, Graham, Lawrence, & Zhang, 2019.

The causes of inter-individual variation in white matter microstructure are not fully understood, but likely involve a complex interplay between genetic and environmental factors over the lifespan. Evidence from both adults and neonates, for instance, suggests that the microstructure of the fornix is highly heritable (Budisavljevic et al., 2016, Lee et al., 2015). The fornix is also one the earliest white matter tracts to mature, reaching its peak FA and minimum MD before age 20 (Lebel et al., 2012), and potentially nearing maturation during infancy and childhood (Dubois et al., 2008). At the same time, evidence suggests that fornix microstructure displays learning-related plasticity, even over short time periods. For instance, short-term spatial learning, in both rodents and humans, has been shown to induce alterations in diffusion indices of fornix microstructure (Hofstetter, Tavor, & Tzur-Moryosef, 2013). Similarly, navigational ability is influenced by both genetic factors and experience Coutrot et al., 2018, Konishi et al., 2016, Lee and Spelke, 2010. Thus, fornix microstructure is likely to both shape, and be shaped by spatial navigation, in a bidirectional fashion (Bechler et al., 2018).

To conclude, by modelling learning performance on a virtual-reality ‘water maze’, we found that the microstructure of the main white matter pathway linking the hippocampus with medial prefrontal cortex and medial diencephalon – the fornix – predicted individual differences in human flexible navigational learning. These results suggest that a full understanding of the biological underpinnings of inter-individual differences in human navigational ability requires not only the analysis of local brain structures, but of a distributed “extended navigation system”, underpinned by white matter fibre pathways. Critically, given the vulnerability of this brain system to the deleterious effects of aging (Lester, Moffat, Wiener, Barnes, & Wolbers, 2017), but also pathology in Alzheimer's disease (Braak and Braak, 1991, Oishi and Lyketsos, 2014), it is a key priority to develop behavioural markers of navigational ability that are sensitive to inter-individual variation in this network, as seen here. One study in rodents, for instance, found that poorer learning on the MWM in early life predicted cognitive impairment in later life, but also that extensive training in poorer learners buffered against age-related learning impairments (Hullinger & Burger, 2015). Studies such as this highlight the potential of navigational learning, particularly as assessed using translation paradigms (Possin et al., 2016), for characterising, and potentially ameliorating (Clemenson, Henningfield, & Stark, 2019), the effects of cognitive decline.

## Research data

No part of the study procedures or analyses was pre-registered prior to the research being conducted. We report how we determined our sample size, all data exclusions (if any), all inclusion/exclusion criteria, whether inclusion/exclusion criteria were established prior to data analysis, all manipulations, and all measures in the study. The raw neuroimaging data cannot be shared publicly due to ethical restrictions relating to General Data Protection Regulation. Data will be released to researchers on the following conditions: approval from the local ethics committee and with appropriate safeguards to protect from identification of individuals. The experimental task, anonymous derived data (e.g., diffusion tensor metrics, latency data), and analysis scripts/code have been made available via the Open Science Framework and can be accessed at https://osf.io/np7c8/. Any questions and additional requests for data can be sent to the corresponding author via email.

## Open practices

The study in this article earned an Open Materials badge for transparent practices. Materials for the study are available at https://osf.io/np7c8/?view_only=0c0b276017d34eb4a1e959061bb32047.

## CRediT authorship contribution statement

**Carl J. Hodgetts:** Conceptualization, Formal analysis, Data curation, Visualization, Writing - original draft. **Martina Stefani:** Conceptualization, Investigation, Formal analysis, Writing - review & editing. **Angharad N. Williams:** Formal analysis, Writing - review & editing. **Branden S. Kolarik:** Resources, Software, Formal analysis, Writing - review & editing. **Andrew P. Yonelinas:** Resources, Software, Writing - review & editing. **Arne D. Ekstrom:** Resources, Software, Writing - review & editing. **Andrew D. Lawrence:** Conceptualization, Supervision, Writing - review & editing. **Jiaxiang Zhang:** Formal analysis, Writing - review & editing. **Kim S. Graham:** Conceptualization, Funding acquisition, Supervision, Writing - review & editing.

## Declaration of Competing Interest

None.
